# Spectral changes associated with transmission of OLED emission through human skin

**DOI:** 10.1038/s41598-019-45867-9

**Published:** 2019-07-08

**Authors:** Soniya D. Yambem, Trent L. Brooks-Richards, David P. Forrestal, Marcin Kielar, Pankaj Sah, Ajay K. Pandey, Maria A. Woodruff

**Affiliations:** 1 School of Chemistry Physics and Mechanical Engineering, Science and Engineering Faculty, Queensland University of Technology (QUT), Brisbane, QLD 4000 Australia; 2 Institute of Health and Biomedical Innovation, Queensland University of Technology (QUT), Kelvin Grove, Queensland 4059 Australia; 3 Queensland Brain Institute, The University of Queensland, St Lucia, QLD 4072 Australia; 4 School of Electrical Engineering and Computer Science, Science and Engineering Faculty, Queensland University of Technology (QUT), Brisbane, QLD 4000 Australia

**Keywords:** Organic LEDs, Applied optics

## Abstract

A recent and emerging application of organic light emitting diodes (OLEDs) is in wearable technologies as they are flexible, stretchable and have uniform illumination over a large area. In such applications, transmission of OLED emission through skin is an important part and therefore, understanding spectral changes associated with transmission of OLED emission through human skin is crucial. Here, we report results on transmission of OLED emission through human skin samples for yellow and red emitting OLEDs. We found that the intensity of transmitted light varies depending on the site from where the skin samples are taken. Additionally, we show that the amount of transmitted light reduces by ~ 35–40% when edge emissions from the OLEDs are blocked by a mask exposing only the light emitting area of the OLED. Further, the emission/electroluminescence spectra of the OLEDs widen significantly upon passing through skin and the full width at half maximum increases by >20 nm and >15 nm for yellow and red OLEDs, respectively. For comparison, emission profile and intensities of transmitted light for yellow and red inorganic LEDs are also presented. Our results are highly relevant for the rapidly expanding area of non-invasive wearable technologies that use organic optoelectronic devices for sensing.

## Introduction

While applications of organic light emitting diodes (OLEDs) in display and lighting technologies have reached the commercial market, a current and promising trend in application of OLEDs is in wearable technology^1,2^. This emerging trend of using OLEDs in wearable technology is due to the unique property of OLEDs in being highly flexible and stretchable^3–5^, and therefore, having the potential to conform to any shape and size of the human body. OLEDs are also very thin and light weight and could be fabricated with simple solution processing or ink-jet printing methods^6–8^. Further, OLED emission is easily tuneable and therefore, is particularly effective as a light source. Another major advantage of OLEDs is homogenous illumination over a large area^9^, making it a suitable light source for optical sensing with small implants which tend to move with time leading to misalignment of sensing elements. The printability of OLEDs also opens up possibilities to design integrated complex and compact devices.

Applications of OLEDs in wearable technology are mainly as a light source where emission from an OLED needs to penetrate past the skin barrier^1,2^. In particular, accurate measurements have been reported for cutaneous sensors with OLEDs, such as pulse oximetry. Such applications require OLED light to penetrate the skin, which is a highly inhomogeneous medium with a very complex multi-layered structure. Optical properties of human skin such as reflection, absorption and scattering have been reported earlier^10–13^. It is well known that, even within the visible light wavelengths, longer wavelengths penetrate deeper than shorter wavelengths. Additionally, highly coherent and collimated beams of light, such as light provided by a laser, have deeper penetration in tissues as compared to incoherent sources^14^. On the other hand, light from OLEDs are highly incoherent and non-monochromatic, and OLEDs have a wide electroluminescence (EL) spectrum. Recently, we reported dynamic colour tuning of OLED emission by using an anisotropic thin film filter^15^. However, to realise the full potential of OLEDs in biometric and therapeutic applications, understanding and quantification of transmission of OLED emission through skin is essential.

In this report, we describe the changes in intensity and EL spectrum of emission from yellow and red OLEDs when it penetrates human skin. We find that the intensity of emission reduces and the EL spectrum widens significantly, as it passes through skin. Reduction in intensity is obvious since skin absorbs, reflects and scatters light. However, the significant widening in EL spectrum is seen only for OLED emissions. We also compared our results with conventional LEDs. These results provide guidelines for the application of OLEDs in wearable technologies.

## Results

**Figure 1:**
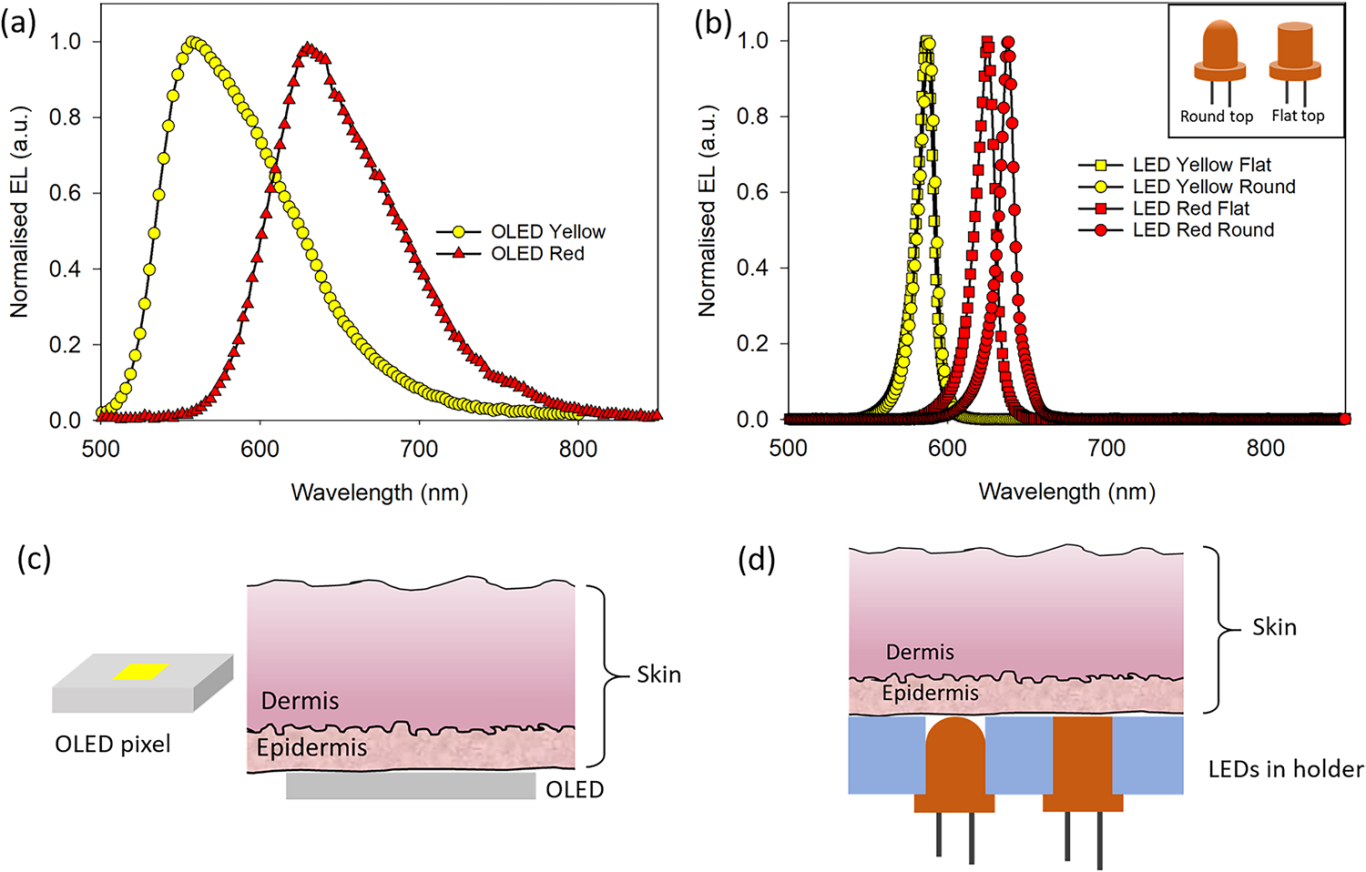
(**a**) Electroluminescence spectra of yellow and red OLEDs. (**b**) Electroluminescence spectra of yellow and red LEDs with rounded/dome and flat tops. Inset: Schematic of dome and flat tops of LED. (**c**) Schematic of an OLED pixel on substrate and OLED emission passing through skin. (**d**) Schematic of LEDs in contact with skin in the experimental set-up.

Simple solution processed OLEDs with yellow and red emissions were used for this study. The structure of OLEDs along with materials used and current-voltage-luminance characteristics are provided in Fig. S1. The EL spectra of the OLEDs are shown in Fig. 1a. The EL of both yellow and red OLEDs has a full width half maximum (FWHM) of around 90 nm, which is typical of fluorescent OLEDs^15,16^. EL spectra of yellow and red commercial LEDs used for comparative study are shown in Fig. 1b. LEDs with two types of encasing, a rounded top and a flat top, were used (inset of Fig. 1b). A flat top LED will have an emission profile closer to that of an OLED, which has a Lambertian emission profile^15^. The ELs of yellow flat (YF) and yellow round (YR) LEDs are almost exactly the same, whereas EL of red round (RR) LED is red-shifted as compared to red flat (RF) LED. For both red and yellow LEDs, the FWHM is ~10–14 nm, which is considerably narrower in comparison to FWHM of OLEDs. To investigate changes in EL spectrum and intensity when OLED light passes through skin, the human skin sample is placed directly on top of the OLEDs, which has a flat surface (Fig. 1c). For LEDs, a holder was designed to ensure that only the top of the LEDs are in contact with the skin, while ensuring the skin stays flat (Fig. 1d). Details of the experimental set up can be found in Fig. S2.

### Changes in intensity of OLED emission

**Figure 2:**
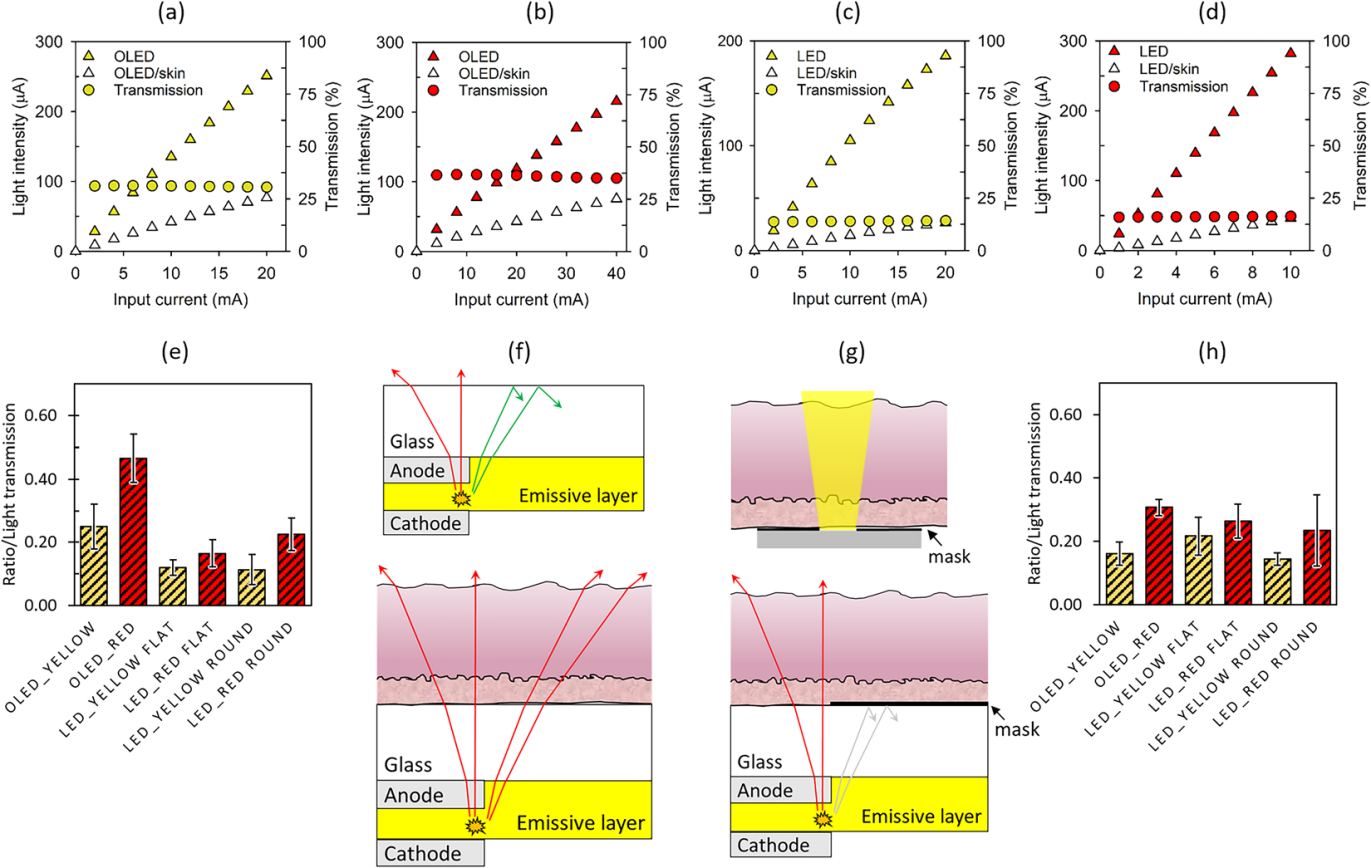
Intensity of emission, emission through skin and percentage of transmission through skin as a function of applied current for (**a**) yellow OLED, (**b**) red OLED, (**c**) YF LED and (**d**), RF LED. (**e**) Percentage of light transmitted through skin for OLEDs and LEDs for skin samples from anterior wrist. (**f**) Ray traces of emission at the edges of OLED pixel with and without skin. (**g**) Skin on top of a masked OLED and ray traces of emission at the edges of a masked OLED pixel with skin. (**h**) Percentage of light transmitted through skin for masked OLEDs and LEDs for skin samples from inner elbow.

Figure 2a,b show typical emission intensity and intensity of emission through skin of yellow and red OLEDs, respectively, as a function of input current of the OLED. The intensity of emission is presented as current of the photodiode that was used in the measurement. The corresponding luminance and voltage for the input currents used can be seen from the current-voltage-luminance characteristics shown in Fig. S1. As seen in Fig. 2a,b, the intensity of OLED emission increases almost linearly with input current, a typical characteristic of OLEDs^17^. The intensity of OLED emission through skin also varies linearly with applied current, indicating that the percentage of OLED light transmission through skin is independent of light intensity. As such, percentage of OLED light transmitted through skin is constant for all intensities of emission tested (Fig. 1a), which is consistent with an earlier report showing that light penetration depth is independent of light intensity^18^. Like OLEDs, similar characteristics of linear increase in intensity of emission and emission through skin, and constant percentage of transmitted light were seen for LEDs, as shown in Fig. 2c,d for YF LED and RF LED, respectively. The characteristics for YR and RR LEDs are included in Fig. S3. The intensities of light emission tested for LEDs are kept in the same range as for OLEDs (Fig. 2a–d, Fig. S3).

Average percentage of light transmitted for skin samples taken from the anterior wrist are shown in Fig. 2e, with standard deviation as error. The average was calculated over various intensities of OLED emission and across all points tested on the skin samples. For yellow OLEDs, ~25% of emitted light is transmitted through skin while for red OLEDs ~46% of emission gets transmitted (exact values are provided in Table S1). The higher transmission of light for red OLED is expected since longer wavelengths are known to have better penetration through human tissues^19^. The same trend is also seen for transmission of emission from LEDs. However, the percentage of transmission for OLEDs is almost double compared to LEDs (Fig. 2e, Table S1) for both yellow and red, even though LEDs have a higher coherence length and are thus expected to have better penetration through skin^20,21^. OLEDs have a coherence length shorter than 2 μm whereas LEDs have a coherence length of ~12 μm^20,21^. Computational methods have shown that beam-width of light sources plays a role in depth of tissue penetration^19^, which means a bigger beam size will result in a higher transmission value. That being said, the difference in beam size of OLEDs (10 mm^2^) and LEDs (7 mm^2^) is not large enough to make a difference in transmission as significant as that seen in our study. It is also unlikely for the skin to form a resonant cavity with the OLED and contribute to enhancement in transmission because skin has very low reflection of ~5–7% and is not a very smooth surface^13^. A possible cause for the higher transmission for OLEDs is the skin acting as a light extraction layer for emission at the edge of the OLED pixel, demonstrated by ray diagrams in Fig. 2f. Since skin has a lower refractive index (<1.5)^22^ compared to glass (>1.5), some of the rays (in green) which would otherwise be reflected back inside the OLED are out-coupled by skin, which is in direct contact with the glass substrate. Similar observations of out-coupling edge emissions in OLEDs have been reported when light extracting microlens arrays were used on OLEDs, which led to blurry edges of OLED pixels^23^. To investigate if out-coupling of the edge emissions is the cause of the higher transmission of OLED emission as compared to LEDs, we repeated the experiment with a masked OLED using skin samples from the inner elbow. A mask of the exact dimensions as the OLED pixel was used to block the rays at the edge from coming in contact with skin, as shown in Fig. 2g. The average transmissions of masked OLEDs and LEDs are shown in Fig. 2h. The transmission for masked OLEDs through skin are within the same range as transmission for LEDs for both colours, which confirms that high transmission for unmasked OLEDs (Fig. 2e) is mainly due to out-coupling of edge emissions from the OLEDs to skin. For LEDs, out-coupling of edge emission is absent, since the emissions coming out of the sides are blocked by the LED holder (Fig. S4) and the skin is not in touch with the sides of the LEDs in the experimental set-up.

To find the difference in the amount of transmitted emission for masked and unmasked OLEDs, we repeated the experiment for both masked and unmasked OLEDs on the same skin samples which were from the shoulder. The results are shown in Fig. S5 and Table S1. Compared to unmasked OLEDs, there is a ~35% and ~40% drop in the amount of transmitted light for masked yellow and red OLEDs, respectively, indicating the strong out-coupling of edge emissions of OLEDs to skin. As before, the transmitted amounts of light for masked OLEDs are in the same range as transmitted amount for LEDs. Further, looking at the transmission intensities for all skin samples in Table S1, the transmission intensities for OLEDs are higher for skin samples from the shoulder. This is most likely due to the slightly thinner skin samples from the shoulders (Table S2). There is no major contribution in the difference in transmitted intensities from variation in skin pigmentation since the skin samples have no major difference in pigmentation (Fig. S6).

### Changes in EL of OLED

**Figure 3:**
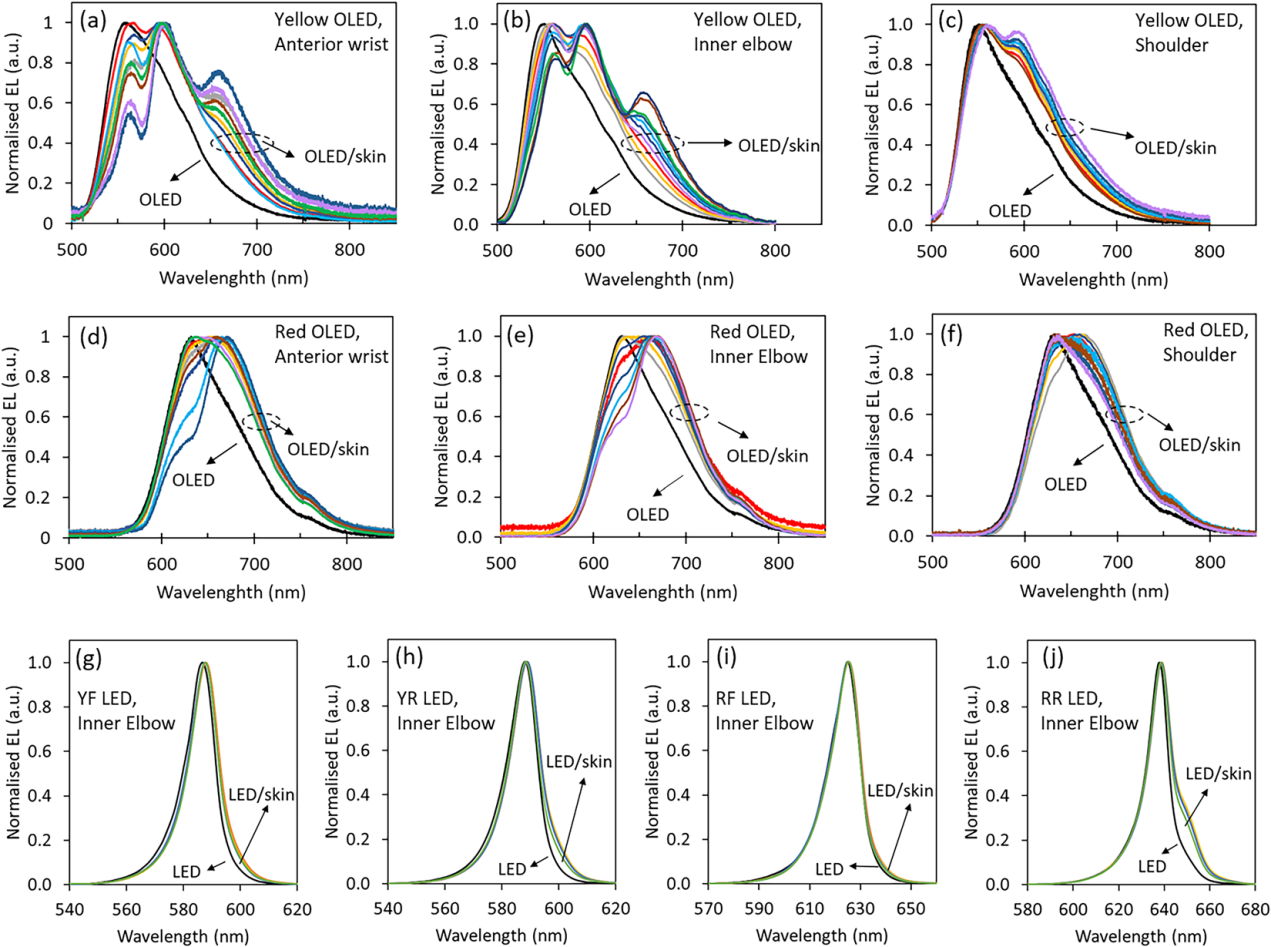
Changes in EL spectra of OLEDs and LEDs as it passes through skin. EL spectra of bare OLEDs and LEDs are in black and coloured plots represent EL spectra of OLEDs and LEDs through skin. (**a**–**c**) EL spectrum of the yellow OLED and changes in the spectrum as it passes through skin samples from anterior wrist, inner elbow and shoulder, respectively. (**d**,**e**) EL spectrum of the red OLED and changes in the spectrum as it passes through skin samples from anterior wrist, inner elbow and shoulder, respectively. (**g**–**j**) EL spectrum of LED and changes in the EL spectrum after passing through skin samples from inner elbow for YF, YR, RF, RR LEDs respectively.

In addition to changes in intensity of OLED emission through skin, changes in EL spectra were also studied. Figure 3a–c, shows EL spectra of yellow OLEDs through skin samples from anterior wrist, inner elbow and shoulder, respectively. These EL spectra are representative of the changes observed after passing through the skin samples. A complete set of EL spectra for all points of skin samples tested are provided in Fig. S7. While yellow OLED emission has one single peak at ~550 nm, once it passes through skin, two or more dominant peaks appear at wavelength ranges of ~555–570 nm, ~590–600 nm and ~650–660 nm. The significant change in the spectra as it passes through skin is because absorption and scattering coefficients of skin changes significantly across the visible spectrum. From Fig. 3, we also see that across all skin samples from the three different regions, the EL spectrum broadens as the light passes through skin. However, the extent of widening and appearance of dominant peaks are different for skin samples from different regions. Such a difference is expected since skin differs from one region to the other in terms of tissue material contents and structures^24^. Broadening of EL spectrum is also seen for red OLEDs once it passes through skin, as shown in Fig. 3d–f. The peak emission wavelength for red OLED is ~630 nm. Unlike for yellow OLEDs, there is only one peak in the EL for red OLED once it passes through skin. In some cases, the peak of EL occurs at wavelength range of ~630–640 nm, which is very close to the peak emission of the OLED itself and in others, the peak shifts to a longer wavelength between ~650–670 nm. In all cases, the EL spectrum widens as compared to the normal OLED emission. On the other hand, there is very little to no change in the EL spectra for commercial LEDs once it passes through skin, as shown in Fig. 3g–j for skin samples from inner elbow. ELs through skins samples from inner wrist and shoulder are presented in Fig. S8. The minute change in the EL for commercial LEDs through skin is most likely due to the very narrow emission bandwidth of LEDs as compared to OLEDs (see Fig. 1a,b).

**Figure 4:**
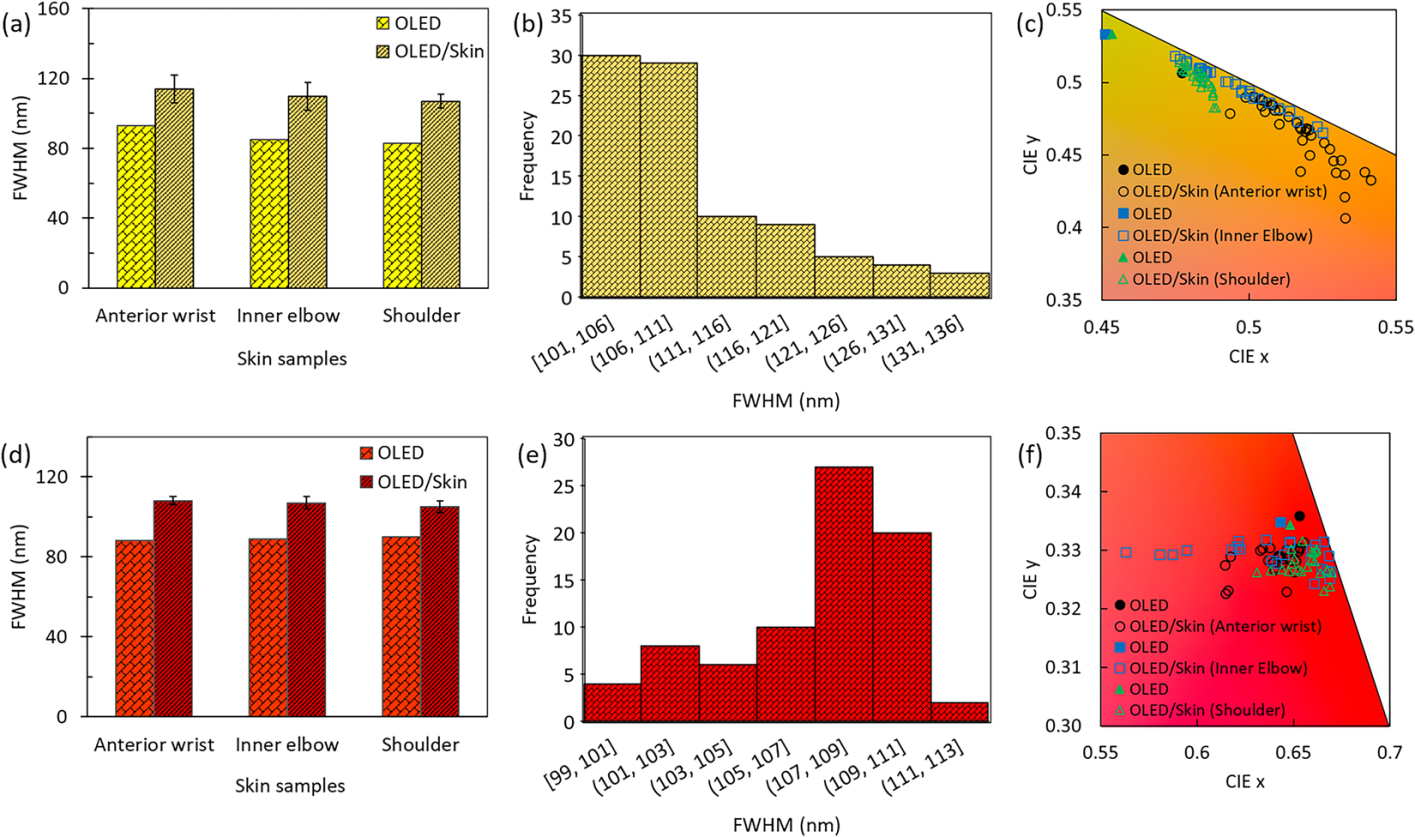
FWHM of OLED emission and OLED emission after passing through skin samples for (**a**) yellow and (**d**) red OLEDs for skin samples from three different regions of the human body. Distribution of FWHM of emissions through skin samples for all skin types combined for (**b**) yellow and (**e**) red OLEDs. Chromaticity plots of OLED emissions and emissions through skin for (**c**) yellow and (**f**) red OLEDs.

The extent of widening of the EL spectra of OLEDs upon passing through skin can be seen more clearly and quantified through the change in FWHM of the emission spectra. The FWHM of OLED emission and average FWHM once it passes through skin are shown in Fig. 4a,d for yellow and red OLEDs, respectively. Exact values are provided in Table S3. Once the emission passes through skin, there is an increase in FWHM for yellow OLEDs by an average of ~21–25 nm, averaged over all skin types. The increase in FWHM is slightly less for red OLEDs, averaging ~15–19 nm. The frequency distribution of FWHM of ELs through skin for all skin samples combined are shown in Fig. 4b,e for yellow and red OLEDs, respectively. The distribution is left skewed for yellow OLEDs and right skewed for red OLEDs, indicating median FWHM is less than average FWHM for yellow OLEDs and more for red OLEDs. The effect of widening of EL spectra of OLED on chromaticity once it passes through skin is shown in Fig. 4c,f for yellow and red OLEDs, respectively. The CIE coordinates for yellow OLEDs shift towards orange as a result of widening of the EL. There is almost equivalent shift in both x and y coordinates of CIE. For red OLEDs, the shift in x co-ordinate is more than the shift in the y coordinate. Insignificant changes in chromaticity were observed for commercial LEDs, which is evident from the very small changes in EL after passing through skin (Fig. 3g–j).

## Discussion

The results for changes in EL spectrum after passing through skin shows that the width of EL spectrum is an important parameter to be considered while designing optoelectronic devices for wearable electronics. The narrower the width of EL spectrum, the less the change in the shape and width of EL spectrum after passing through skin. Both broad and narrow spectra have their niche applications. Whilst a broad spectrum is useful for comparison of signals at different wavelengths, such as sensing of blood oxygenation levels and heart rate, narrow spectrums will be desirable for targeting specific excitation. For example, excitations of implants below the skin for release of a specific chemical, such as a drug or a hormone. Shaping and narrowing of OLED emissions is achieved by incorporating a microcavity in the OLED and also through variation in thickness of the emissive layer. More recently, we have shown that FWHM of OLED emission can be reduced by using organic thin film filters^15^. Further development of spectral narrowing techniques of OLEDs will lead to an increase in the applicability of OLEDs in optoelectronic wearable devices and sensors.

In summary, we report our findings on transmission of OLED light through human skin for yellow and red OLEDs. The intensity attenuation of OLED light passing through skin is comparable with light from commercial LEDs passing through skin. If the OLEDs are not masked, edge emissions coupling to skin leads to higher intensity of OLED light going through skin. ELs of OLEDs widen significantly upon passing through skin, making considerable changes in chromaticity co-ordinates. Our findings are very topical and relevant with the current trend in OLED research for applications in wearable electronics as a light source.

## Methods

### OLED fabrication and testing

Bottom emitting yellow and red OLEDs were fabricated using standard processes reported earlier^15,16^. Current-voltage-brightness measurements were recorded using a Keysight B2901A sourcemeter and a photodiode calibrated with a Konica Minolta CS 200 luminance meter. EL spectra of the OLEDs were measured using an Ocean Optics spectrometer (USB4000).

### Preparation of skin samples

Sections of skin from the anterior wrist, inner elbow and shoulder regions taken from cadaveric donors were resected at the hypodermal layer and preserved at ~−20 °C until experimentation. The data presented here is for skin samples taken from one donor body to avoid major variations in skin pigmentation of different individuals. During experimentation, skin was thawed under refrigeration at ~4 °C, cut into ~1.5 cm × 1.5 cm segments and phosphate-buffered saline (PBS pH 7.4) was applied to prevent drying of the tissue. All skin samples were prepared and stored in the same condition during experiments and storage to maintain the same condition for hydration of skin.

Cadaveric skin samples were resected from donors at the Medical Engineering and Research Facility (MERF, QUT). Harvesting and use of cadaveric skin samples was supported by University Human Research Ethics Committee at Queensland University of Technology (QUT) (ethics approval number: 1600000449). The tissues were obtained through QUT’s body bequest program from donors who provided consent for use of their body for the advancement of science and medicine. All experiments were conducted in accordance with the guidelines provided by University Human Research Ethics Committee at QUT.

### Skin thickness and pigmentation measurement

The thickness of the skin samples were measured using thin slices of skin placed under a microscope (Nikon SMZ745T). Skin pigmentation were measured using a skin tone matching device, Pantone x-rite RM200, and matched with skin tones from Pantone Skintone library.

### Light transmission through skin samples

The skin samples were placed directly on the OLEDs and LEDs as illustrated in Fig. 1. Experimental set-up is shown in Fig. S2. The OLEDs and LEDs were driven by current using a Keysight B2962A sourcemeter. The light output of LEDs and OLEDs, with and without skin on top were recorded using a calibrated photodiode. Since the photocurrent of the photodiode increases linearly with the incident light, it allows to measure OLED intensities indirectly. ELs were collected using an Ocean Optics spectrometer USB 4000. The photodiode and optical fiber were always maintained at a fixed distance of 3 mm. This ensures that the skin is not in direct contact with either the photodiode or the optical fiber. For experiments with masked OLEDs, either a black tape (thickness = 300 μm) or stainless steel mask (thickness = 150 μm) was used to mask the OLEDs.

All measurements, OLED characterization and skin related measurements were done at ambient environment.

## Supplementary information

Spectral changes associated with transmission of OLED emission through human skin

## Data Availability

Data supporting the result of our study are available from the corresponding author upon request.
