# Supplementary material for: Spectral changes associated with transmission of OLED emission through human skin
Source: Sci Rep. 2019 Jul 8;9:9875. doi: 10.1038/s41598-019-45867-9 (PMC6614498; doi:10.1038/s41598-019-45867-9)
Supplement: Supplementary file 1 — Spectral changes associated with transmission of OLED emission through human skin [file 41598_2019_45867_MOESM1_ESM.docx]

**Supplementary Information**

**Spectral changes associated with transmission of OLED emission through human skin**

**Soniya D. Yambem^1, 2,^*, Trent L. Brooks-Richards^1, 2^, David P. Forrestal^1, 2^, Marcin Kielar^3^, Pankaj Sah^3^, Ajay K. Pandey^2, 4^, Maria A. Woodruff^1,2^**

^1^School of Chemistry Physics and Mechanical Engineering, Science and Engineering Faculty, Queensland University of Technology (QUT), Brisbane QLD 4000, Australia

^2^Institute of Health and Biomedical Innovation, Queensland University of Technology (QUT), Kelvin Grove, Queensland 4059, Australia

^3^Queensland Brain Institute, The University of Queensland, St Lucia, QLD 4072, Australia

^4^School of Electrical Engineering and Computer Science, Science and Engineering Faculty, Queensland University of Technology (QUT), Brisbane QLD 4000, Australia

*[soniya.yambem@qut.edu.au](mailto:soniya.yambem@qut.edu.au)

The structure of OLEDs fabricated for this study is shown in Figure S1a. Current and luminance as a function of voltage for yellow and red OLEDs are shown in Figures S1b and S1c, respectively.


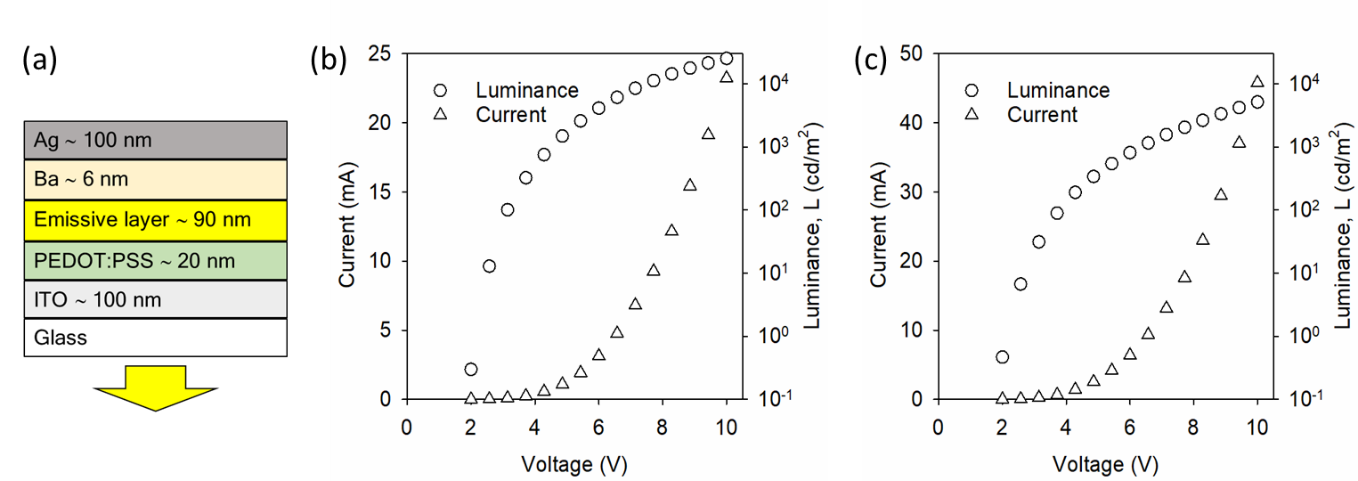


**Figure S1.** (a) Structure of OLEDs used in this study. The emissive layer is either polymer Super Yellow for yellow OLEDs or polymer red for red OLEDs. Current and luminance with respect to voltage for (a) yellow and (b) red OLEDs.


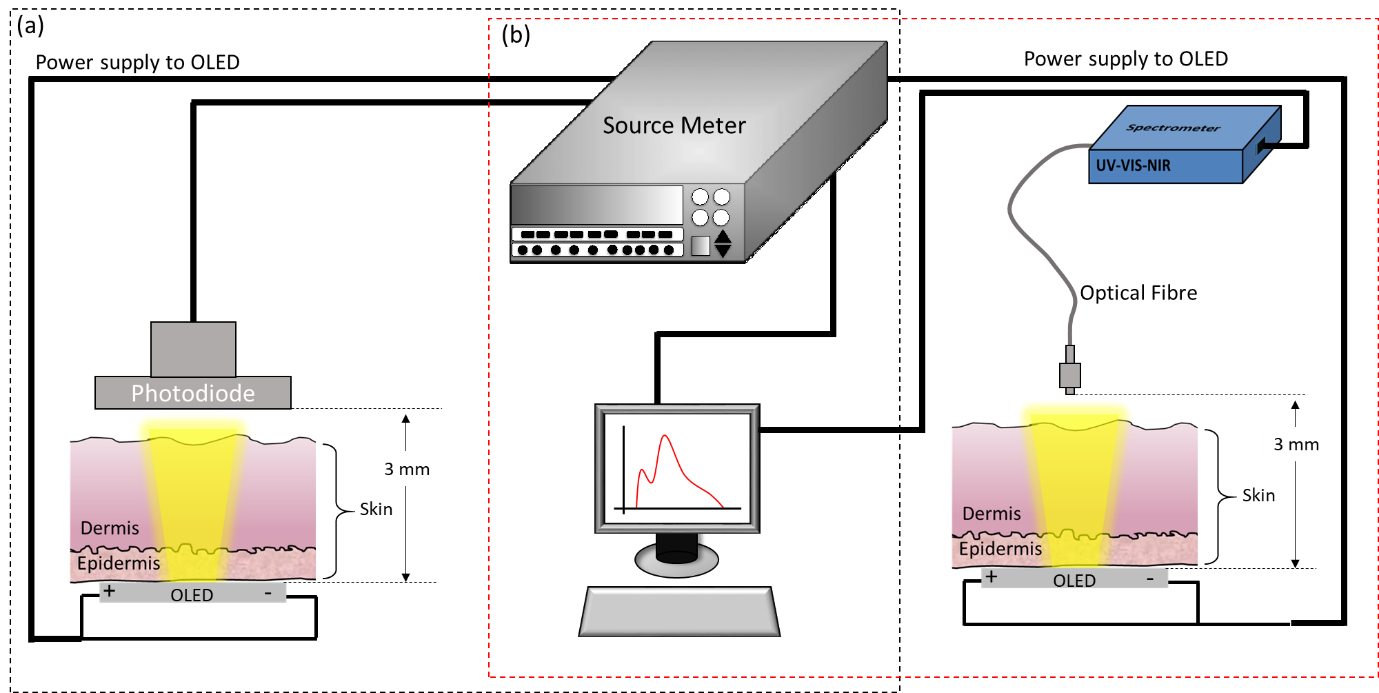


**Figure S2**. (a) Experimental set up for (a) measuring the intensity of light through skin and (b) change in EL spectrum.


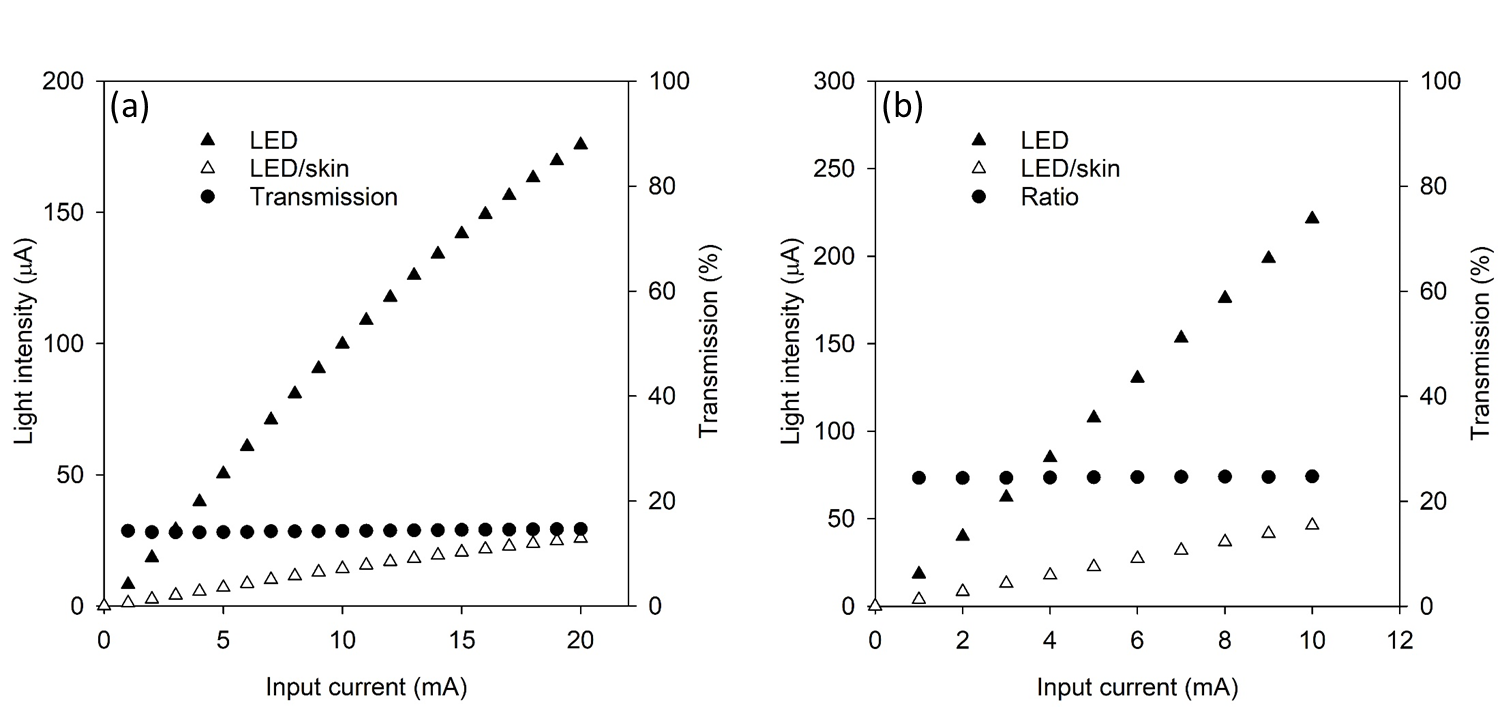


**Figure S3.** (a) Intensity of emission, emission through skin and ratio of the two as a function of applied current for (a) YR LED and (b) RR LED.

**Table S1.** Amount of light transmitted for OLEDs and LEDs. Standard deviations are provided as errors.

|  | **Transmission (%)** | | | |
| --- | --- | --- | --- | --- |
| **OLED/LED** | **Anterior Wrist** | **Inner Elbow**  **(Masked OLEDs)** | **Shoulder** | **Shoulder**  **(Masked OLEDs)** |
| Yellow OLED | 25 ± 8 | 16 ± 5 | 41 ± 6 | 27 ± 3 |
| Red OLED | 46 ± 8 | 29 ± 8 | 54 ± 9 | 33 ± 7 |
| YF LED | 12 ± 2 | 22 ± 5 | 19 ± 5 |  |
| RF LED | 16 ± 4 | 26 ± 5 | 23 ± 3 |  |
| YR LED | 11 ± 4 | 14 ± 2 | 16 ± 3 |  |
| RR LED | 23 ±5 | 23 ± 10 | 28 ± 8 |  |


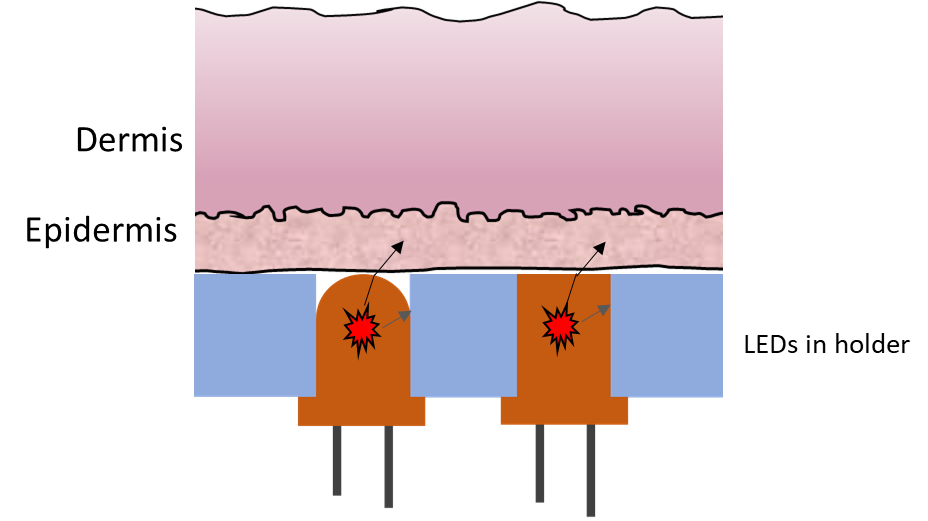


**Figure S4.** Emissions from the sides of the LEDs are blocked by the LED holder and do not contribute to transmission.


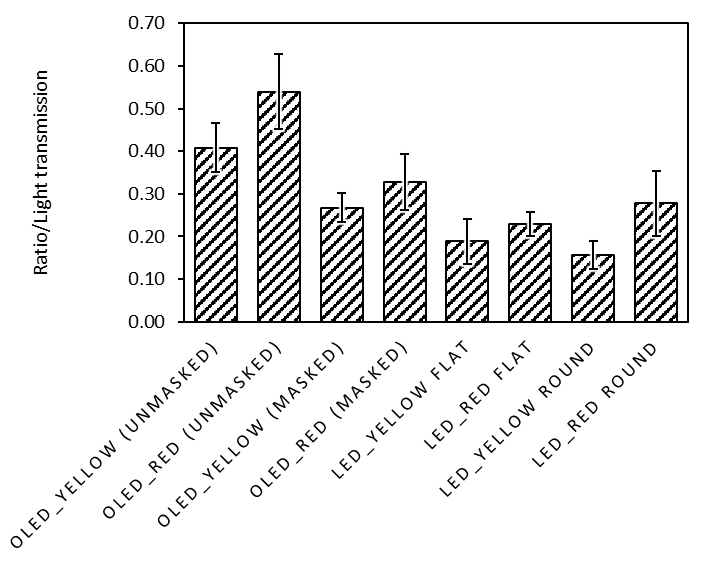


**Figure S5.** Percentage of light transmitted through skin for skin samples from the shoulder for unmasked OLEDs, masked OLEDs, and LEDs.

**Table S2.** Skin thickness for different skin samples. Standard deviations are provided as errors.

| **Skin samples** | Thickness (mm) |
| --- | --- |
| Anterior wrist | 0.95 ± 0.20 |
| Inner Elbow | 0.96 ± 0.32 |
| Shoulder | 0.88 ± 0.27 |


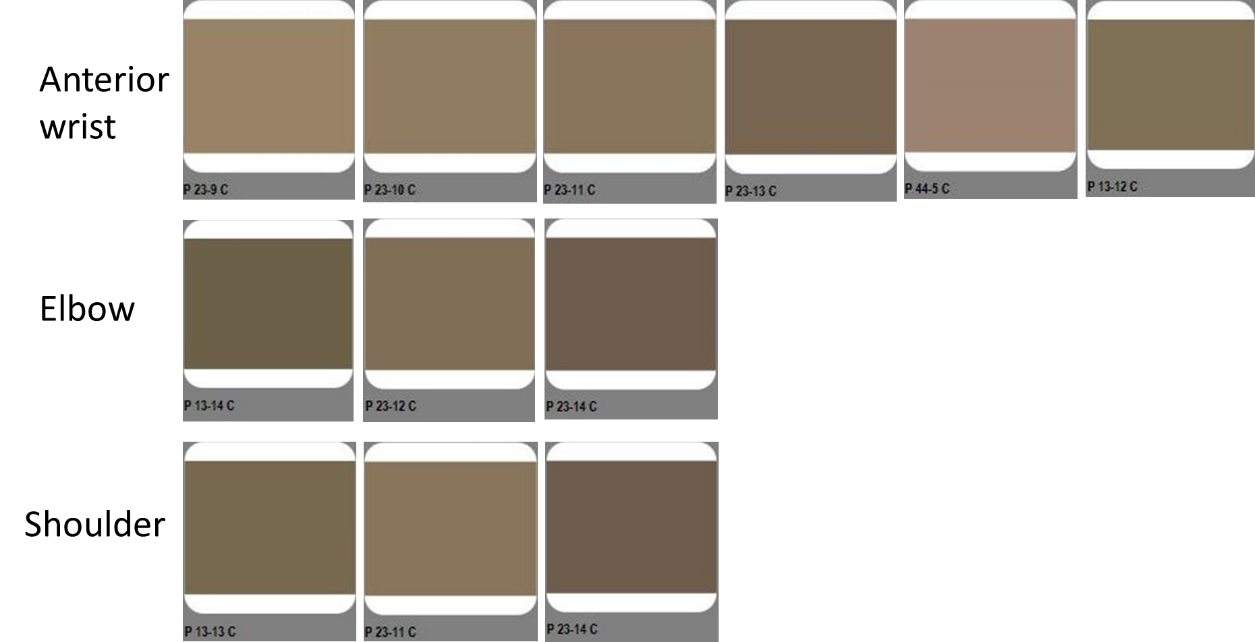


**Figure S6.** Percentage of light transmitted through skin for skin samples from the shoulder for unmasked OLEDs, masked OLEDs, and LEDs.


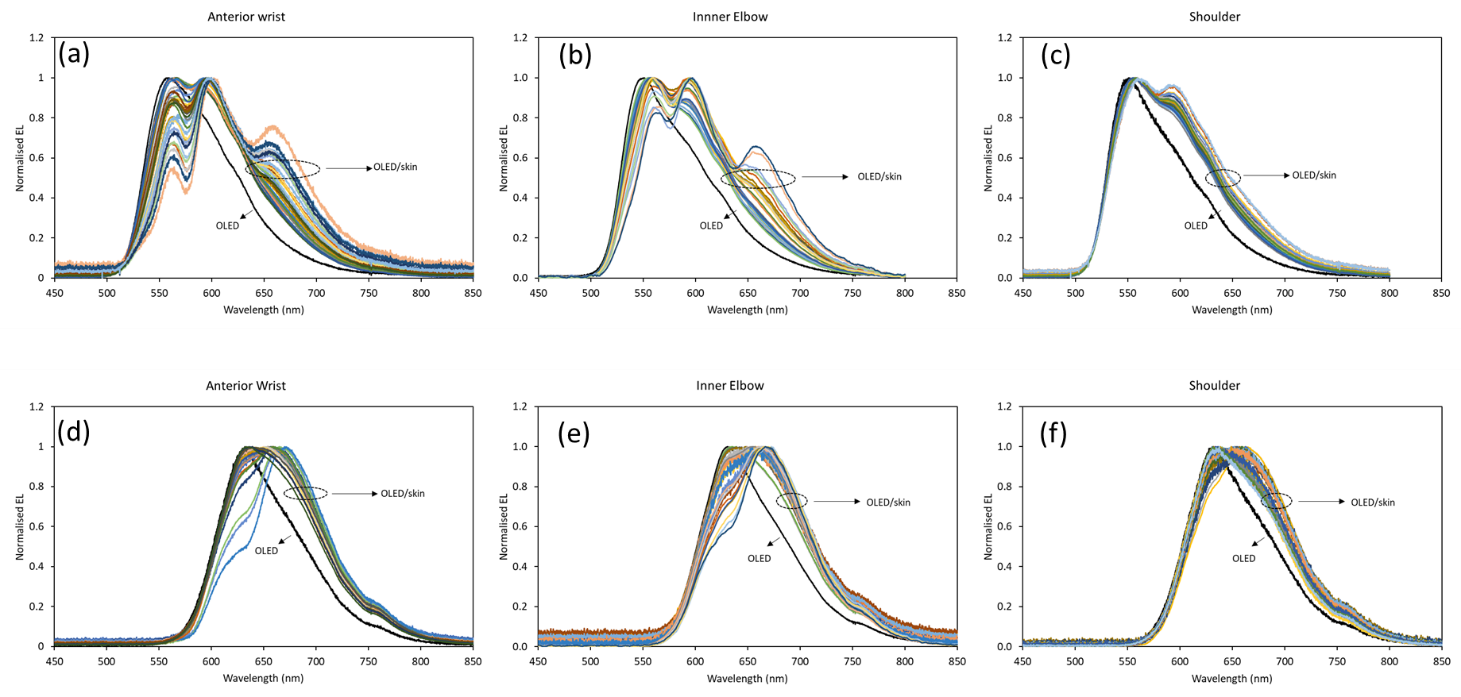


**Figure S7.** ELs of all points measured for yellow OLEDs for skin samples from (a) anterior wrist, (b) inner elbow and (c) shoulder regions. ELs of all points measured for red OLEDs for skin samples from the (d) anterior wrist, (e) inner elbow and (f) shoulder.


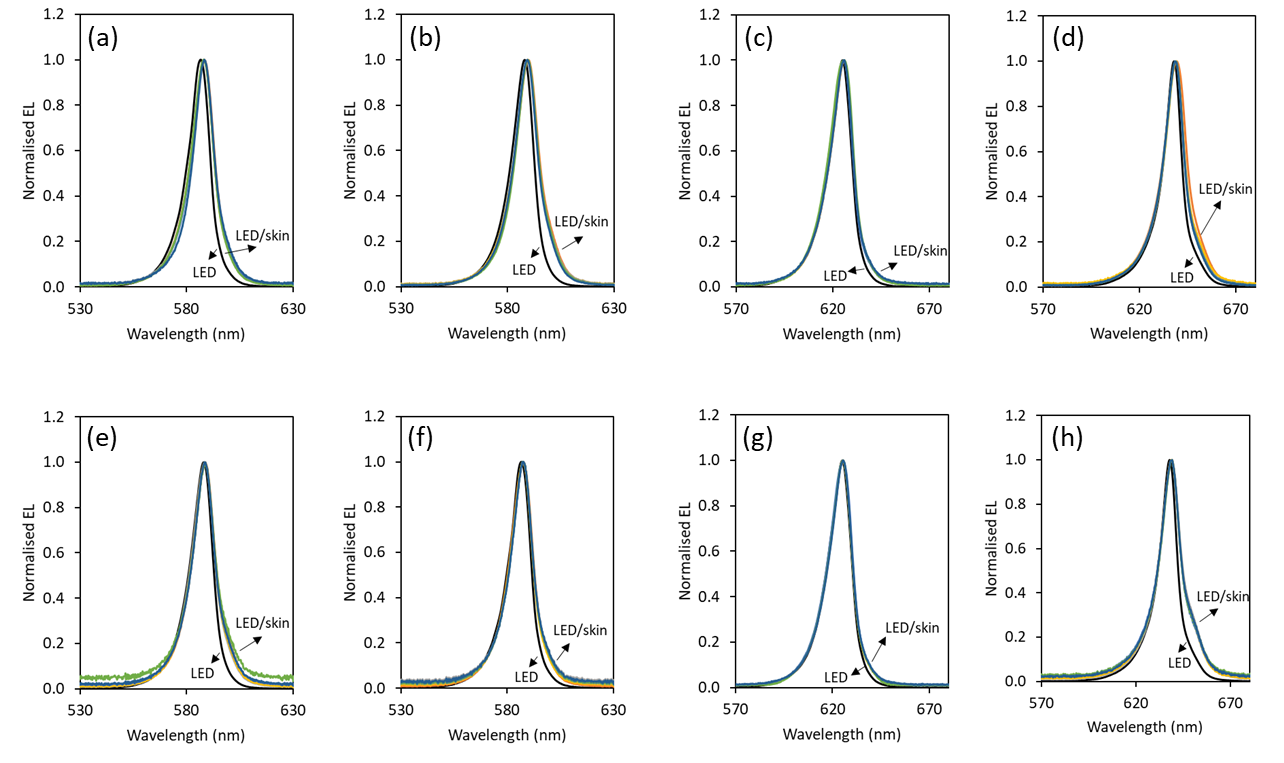


**Figure S8.** ELs of all points measured for YF, YR, RF and RR LEDs, respectively for skin samples from the (a-d) anterior wrist and (e-h) shoulder.

**Table S3.** Full width at half maximum for ELs of OLEDs and through skin for different skin samples and change in FWHM for OLED emissions once it passes through skin.

|  | | **FWHM (nm)** | |
| --- | --- | --- | --- |
| **Skin type** |  | **Yellow OLED** | **Red OLED** |
| Anterior Wrist | OLED | 93 | 88 |
|  | OLED/skin | 114 ± 8 | 107 ± 5 |
|  | ΔFWHM | 21 | 19 |
| Inner Elbow | OLED | 85 | 89 |
|  | OLED/skin | 110 ± 8 | 107 ± 3 |
|  | ΔFWHM | 25 | 18 |
| Shoulder | OLED | 83 | 90 |
|  | OLED/skin | 107 ± 4 | 105 ± 3 |
|  | ΔFWHM | 24 | 15 |
